# Magnetocardiography for Exploratory Risk Stratification in Patients With Three‐Vessel Coronary Artery Disease: A Single‐Center Prospective Cohort Study

**DOI:** 10.1155/cdr/5552371

**Published:** 2026-07-16

**Authors:** Ziyu An, Shipan Wang, Min Zhang, Mingduo Zhang, Lanxin Feng, Shuwen Yang, Qi Zhou, Haiyang Hu, Chenchen Tu, Hongjia Zhang, Xiantao Song

**Affiliations:** ^1^ Department of Cardiology, Beijing Anzhen Hospital, Capital Medical University, Beijing, China, ccmu.edu.cn; ^2^ Department of Cardiovascular Surgery, Beijing Anzhen Hospital, Capital Medical University, Beijing, China, ccmu.edu.cn; ^3^ Beijing Anzhen Hospital, Capital Medical University, Beijing Institute of Heart, Lung, Blood Vessel Diseases, Beijing, China, heartinsti.com; ^4^ Department of Cardiology, Beijing Friendship Hospital, Capital Medical University, Beijing, China, ccmu.edu.cn

**Keywords:** coronary artery disease, magnetocardiography, prognostic, SYNTAX score, three-vessel coronary artery disease

## Abstract

**Background and Aims:**

Patients with three‐vessel coronary artery disease (3V‐CAD) remain at heterogeneous risk for major adverse cardiovascular and cerebrovascular events (MACCE) after revascularization. Magnetocardiography (MCG) is a noncontact and radiation‐free technique that may capture electrophysiological abnormalities not reflected by anatomical risk scores. This study evaluated the exploratory prognostic value of MCG‐derived parameters in patients with 3V‐CAD.

**Methods:**

This single‐center prospective cohort study included 544 patients with 3V‐CAD who underwent coronary revascularization. MCG recordings were obtained before revascularization using a 36‐channel optically pumped magnetometer‐based system. The primary endpoint was MACCE, defined as cardiac death, stroke, myocardial infarction, or unplanned revascularization. Cox regression models were constructed using clinical variables, SYNTAX score, and an MCG composite variable. Model performance was assessed using discrimination, calibration, decision curve analysis, and bootstrap internal validation.

**Results:**

During a median follow‐up of 725 days, 37 patients (6.8%) experienced MACCE. The SYNTAX score was independently associated with MACCE after adjustment for clinical variables and MCG score (hazard ratio [HR], 1.08; 95% confidence interval [CI], 1.04–1.12; *p* < 0.001). The MCG composite variable was also associated with MACCE after adjustment for clinical variables and SYNTAX score (HR, 2.74; 95% CI, 1.57–4.79; *p* < 0.001). Model discrimination increased from 0.596 for clinical variables alone to 0.682 after adding SYNTAX score and to 0.727 after adding MCG. However, the improvement from the clinical + SYNTAX model to the MCG‐added model was not statistically significant (*p* = 0.17). Bootstrap validation showed an optimism‐corrected *C*‐index of 0.672.

**Conclusion:**

MCG‐derived parameters were associated with MACCE and may provide complementary electrophysiological information for exploratory risk stratification in patients with 3V‐CAD after revascularization. Larger multicenter studies with external validation are needed to confirm its complementary prognostic value.

**Trial Registration:**

Chinese Clinical Trial Registry identifier: ChiCTR2200066942

## 1. Introduction

Three‐vessel coronary artery disease (3V‐CAD) refers to significant stenosis (lumen stenosis ≥ 50%) in all three major coronary arteries, namely, the left anterior descending artery, the left circumflex artery, and the right coronary artery. It is one of the most complex and high‐risk subtypes of coronary artery disease (CAD) [1]. Patients with severe three‐vessel disease may experience myocardial ischemia due to blocked coronary blood flow, presenting as stable or unstable angina pectoris [2]. In severe cases, it can progress to myocardial infarction, heart failure, or even sudden cardiac death. Despite the advancements in revascularization strategies, including percutaneous coronary intervention (PCI) and coronary artery bypass grafting (CABG), as well as optimal medical therapy, the prognosis of patients with 3V‐CAD still requires more precise management [3]. For instance, some patients can achieve a good long‐term prognosis after standard treatment, while others still face a high risk of cardiac death, nonfatal myocardial infarction, or unplanned revascularization [3]. This heterogeneity highlights the limitations of current risk stratification methods in identifying high‐risk individuals who may benefit from enhanced monitoring or therapeutic intervention. As emphasized in the European Society of Cardiology (ESC) Guidelines for the Management of Myocardial Ischemia, precise prognostic assessment is crucial for optimizing personalized care and improving long‐term outcomes in patients with complex CAD [4]. Therefore, there is an urgent need to explore new, noninvasive, and clinically feasible prognostic biomarkers to complement existing tools and enhance the risk stratification of patients with 3V‐CAD.

The current tools used to assess the prognosis of 3V‐CAD have inherent limitations. Clinical variables are easy to obtain, but their discriminatory power is limited. The SYNTAX score, as the gold standard anatomical tool for evaluating lesion complexity, cannot provide information on myocardial ischemia or electrophysiological abnormalities; thus, its prognostic value is limited [5]. Invasive functional tests (such as fractional flow reserve [FFR]) can assess ischemia but are not suitable for routine long‐term monitoring. Currently, there is still a lack of a noninvasive, rapid tool to evaluate long‐term prognosis.

Magnetocardiography (MCG) [6], as a novel imaging detection technology, detects myocardial injury by collecting, recording, and imaging the magnetic field generated by the electrical activity of the heart. It has significant advantages such as being noncontact, noninvasive, rapid, having no radiation exposure, and needing no contrast agents. Previous research has confirmed that MCG has significant value in the diagnosis of coronary heart disease [7]. Based on these considerations, this study was aimed at deriving an exploratory MCG composite variable associated with long‐term major adverse cardiovascular and cerebrovascular events (MACCE) in patients with 3V‐CAD undergoing revascularization and to evaluate whether MCG‐derived information could provide complementary prognostic information when combined with clinical variables and the SYNTAX score.

## 2. Methods

### 2.1. Design and Study Population

This was a single‐center prospective observational cohort study conducted at the Coronary Heart Disease Center of Beijing Anzhen Hospital from December 21, 2022, to December 13, 2023. The data were derived from the registered prospective cohort “Diagnosis of Coronary Heart Disease by Atomic Magnetometer Cardiography.” The study protocol was approved by the Ethics Committee of Beijing Anzhen Hospital, Capital Medical University (Approval Number: KS2022054). All participants provided written informed consent before enrollment.

The inclusion criteria for this registered study were (1) aged between 18 and 80 years, with no gender restrictions, and having the ability to move independently; (2) having stenosis of ≥ 50% in all three major coronary arteries and being planned for coronary revascularization within 1 month; and (3) being able to understand the purpose of the study, voluntarily participating, and signing the informed consent form. The exclusion criteria included (1) previous history of PCI, (2) previous history of CABG, (3) having an implanted cardiac pacemaker or drug pump, (4) claustrophobia, (5) heart failure (NYHA Class III or above), (6) old myocardial infarction (myocardial infarction onset > 6 months before enrollment), (7) severe thoracic deformity, (8) congenital heart disease, and (9) other conditions deemed unsuitable for inclusion by the researchers.

**Figure 1:**
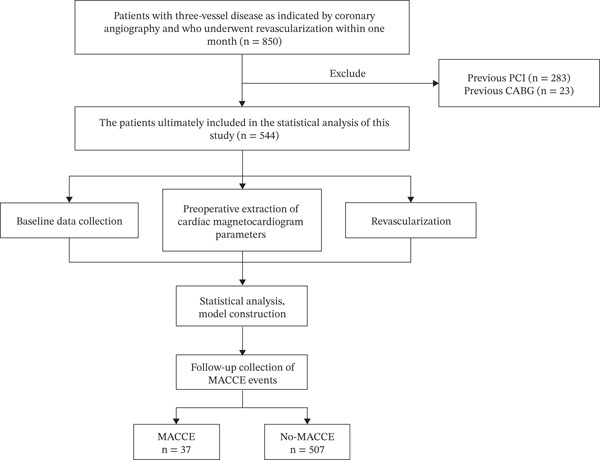
Screening flowchart of the study population. A total of 850 patients with angiographically confirmed three‐vessel coronary artery disease who underwent coronary revascularization within 1 month were screened. After excluding patients with previous PCI (*n* = 283) or previous CABG (*n* = 23), 544 patients were included in the final analysis. Baseline clinical data, pre‐revascularization MCG recordings, and follow‐up MACCE outcomes were collected for model development and evaluation.

A total of 850 patients with three‐vessel disease were screened, and 544 patients were ultimately included. Each patient received the best treatment under the guidance of two experienced cardiologists (either based on PCI or CABG surgery). The study screening process and analysis workflow are shown in Figure 1.

### 2.2. Clinical and Follow‐Up Data Collection

Clinical information was extracted from electronic medical records during hospitalization for each patient. All clinical data were obtained through a standardized review of the hospitals′ electronic medical record systems by trained personnel who were blinded to the study design. The collected variables included demographic characteristics and medical history.

The primary endpoint was MACCE, defined as a composite of cardiac death, stroke, myocardial infarction, and unplanned revascularization. Follow‐up was performed at 1, 6, 12, and 24 months after discharge using outpatient records and telephone interviews. Endpoint events were verified using hospital records and follow‐up information. MCG‐derived research parameters were not used to guide revascularization strategy, postprocedural management, or endpoint assessment. Endpoint events were independently reviewed by two investigators who were blinded to MCG‐derived parameters, and disagreements were resolved by consensus.

### 2.3. MCG System and Recording

A 36‐channel optically pumped magnetometer‐based MCG system (Miracle MCG, Beijing X‐MAGTECH Technologies Ltd., Beijing, China) was used for all MCG recordings. The sensor array was positioned approximately 2 cm above the anterior chest wall while the patient remained in the supine position. Cardiac magnetic field signals were automatically acquired for 90 s using a 6 × 6 sensor array. Before each recording, potential sources of magnetic interference, including metal objects, electronic devices, and magnetic materials, were removed from the measurement environment. MCG detection was performed 1–3 days before coronary revascularization to avoid procedural interference with cardiac electrophysiology.

### 2.4. MCG Data Analysis

MCG data were processed using the system software to reconstruct magnetic field maps and pseudocurrent density maps. These maps were used to characterize the spatiotemporal distribution of cardiac magnetic field signals. A total of 65 candidate MCG parameters related to magnetic field amplitude, current angle, spatial distance, and magnetic pole changes were extracted. Seven exploratory MCG parameters were selected to derive the MCG composite variable: CAg_max_‐Tp, *δ*Dt_min_‐PN, Dt_u1_‐P, Dt_d3_‐N, Dt_d9_‐N, *δ*Ar_min_‐P, and *δ*Ar_min_‐NP. Because individual MCG parameters are not yet standardized clinical biomarkers, they were interpreted as components of an exploratory electrophysiological composite rather than as standalone diagnostic or prognostic markers.

### 2.5. ICA

Coronary angiography (ICA) was conducted during the patients′ hospitalization in accordance with standard procedures, and the degree of coronary stenosis was assessed by experienced interventional cardiologists. All clinical decisions—including the determination of whether to perform revascularization and the selection of revascularization surgical strategies—were made by these experienced interventional cardiologists. Patients who underwent ICA were diagnosed with 3V‐CAD if the stenosis degree of the three major epicardial coronary arteries reached ≥ 50%.

### 2.6. SYNTAX Score

All enrolled patients with CAD underwent ICA, and the SYNTAX score was computed using the SYNTAX score calculator [8]. Two experienced interventional cardiologists were responsible for the SYNTAX score calculation. In cases of discrepancies between the two sets of scoring results, a third experienced interventional cardiologist was consulted to reach a consensus. Patients were stratified into two groups based on their SYNTAX scores: the low‐risk group (SYNTAX score < 22) and the intermediate high‐risk group (SYNTAX score ≥ 22).

### 2.7. Statistical Analysis

All statistical analyses were performed using R software Version 4.3.1 (R Foundation for Statistical Computing, Vienna, Austria). Continuous variables are presented as median and interquartile range (IQR) and were compared using the Mann–Whitney *U* test. Categorical variables are presented as counts and percentages and were compared using Fisher′s exact test or the chi‐square test, as appropriate. All tests were two‐sided, and *p* < 0.05 was considered statistically significant. *p* values were reported according to journal conventions.

The primary endpoint was MACCE during follow‐up. Cox proportional hazard regression was used to assess associations between predictors and MACCE. Three nested models were constructed: Model 1 included clinical variables only; Model 2 included clinical variables and SYNTAX score; and Model 3 included clinical variables, SYNTAX score, and the MCG composite variable. The MCG composite variable was derived from seven standardized MCG parameters using Cox regression and was entered into Model 3 as a single linear predictor. The proportional hazard assumption was assessed using Schoenfeld residuals.

Model discrimination was evaluated using receiver operating characteristic curves, time‐dependent AUC at 730 days, and Harrell′s *C*‐index. Pairwise AUC comparisons were performed using DeLong′s test. Calibration was assessed using calibration plots and Brier scores. Clinical utility was evaluated using decision curve analysis across threshold probabilities of 1%–30%. Internal validation was performed using 1000 bootstrap resamples to estimate the optimism‐corrected *C*‐index. Penalized Cox regression using LASSO was performed as a sensitivity analysis to assess potential overfitting and variable‐selection instability.

The Kaplan–Meier curves were generated to depict event‐free survival, and differences between groups were tested using the log‐rank test. Risk groups were defined using the Youden‐derived cutoff from Model 3, which predicted 730‐day MACCE risk. Subgroup analyses according to SYNTAX score strata were exploratory.

## 3. Results

### 3.1. Patient Characteristics and Clinical Outcomes

A total of 544 patients with 3V‐CAD were included in the final analysis. During a median follow‐up of 725 days, 37 patients (6.8%) experienced MACCE (Figure 1). The baseline characteristics stratified by MACCE occurrence are summarized in Table 1. No missing values were present for variables included in the primary analyses (Table S1). Patients who developed MACCE had a higher prevalence of chronic kidney disease than those without MACCE (10.81% vs. 2.17%; *p* = 0.01) and had higher SYNTAX scores (20.00 [15.00–25.50] vs. 16.00 [11.00–20.00]; *p* = 0.002). Other clinical characteristics, including age, sex, BMI, hypertension, diabetes mellitus, smoking history, LVEF, and clinical presentation, were not significantly different between groups.

**Table 1:** Baseline clinical and MCG characteristics stratified by MACCE status.

Characteristic	Overall (*n* = 544)	No MACCE (*n* = 507)	MACCE (*n* = 37)	*p* value
Age (years)	61.00 [54.00, 67.50]	61.00 [54.00, 67.00]	61.00 [58.00, 68.00]	0.49
Male sex, *n* (%)	403 (74.08%)	377 (74.36%)	26 (70.27%)	0.56
BMI (kg/m^2^)	25.84 [23.81, 27.78]	25.81 [23.83, 27.82]	25.91 [23.80, 27.34]	0.74
Hypertension, *n* (%)	352 (64.71%)	325 (64.10%)	27 (72.97%)	0.37
Diabetes mellitus, *n* (%)	219 (40.26%)	200 (39.45%)	19 (51.35%)	0.17
Smoking history, *n* (%)	120 (22.06%)	114 (22.49%)	6 (16.22%)	0.54
Chronic kidney disease, *n* (%)	15 (2.76%)	11 (2.17%)	4 (10.81%)	0.01
LVEF (%)	63.00 [60.00, 66.00]	63.00 [60.00, 66.00]	63.00 [60.00, 66.00]	0.71
STEMI, *n* (%)	7 (1.29%)	7 (1.38%)	0 (0.00%)	> 0.99
NSTEMI, *n* (%)	33 (6.07%)	31 (6.11%)	2 (5.41%)	> 0.99
Unstable angina, *n* (%)	499 (91.73%)	465 (91.72%)	34 (91.89%)	> 0.99
Stable angina, *n* (%)	3 (0.55%)	2 (0.39%)	1 (2.70%)	0.19
Peripheral arterial disease, *n* (%)	20 (3.68%)	17 (3.35%)	3 (8.11%)	0.15
Hyperlipidemia, *n* (%)	530 (97.43%)	494 (97.44%)	36 (97.30%)	> 0.99
SYNTAX score	16.00 [11.00, 21.00]	16.00 [11.00, 20.00]	20.00 [15.00, 25.50]	0.002
CAg_max_‐Tp	41.54 [22.43, 68.67]	42.09 [22.75, 69.44]	38.66 [−14.04, 55.49]	0.18
*δ*Dt_min_‐PN	−2.43 [−7.63, −0.59]	−2.46 [−7.65, −0.75]	−2.04 [−4.12, 0.44]	0.18
Dt_u1_‐P	−0.95 [−1.00, 0.00]	−0.90 [−1.00, 0.00]	−1.00 [−1.00, 0.00]	0.60
Dt_d3_‐N	484.20 [400.05, 495.00]	485.40 [403.40, 495.00]	467.70 [371.90, 494.20]	0.14
Dt_d9_‐N	−1.00 [−1.00, 48.60]	−1.00 [−1.00, 48.60]	−1.00 [−1.00, 46.40]	> 0.99
*δ*Ar_min_‐P	−1479.50 [−2953.50, −577.00]	−1485.00 [−2961.00, −620.00]	−1400.00 [−2628.00, −559.00]	0.44
*δ*Ar_min_‐NP	−154.00 [−637.50, −15.50]	−153.00 [−641.00, −17.00]	−195.00 [−623.00, −12.00]	0.78

*Note:* Data are presented as median [interquartile range] or *n* (percentage). *p* values were calculated using the Mann–Whitney *U* test for continuous variables and Fisher′s exact test for categorical variables.

Abbreviations: BMI, body mass index; LVEF, left ventricular ejection fraction; MACCE, major adverse cardiovascular and cerebrovascular events; MCG, magnetocardiography; NSTEMI, non‐ST‐segment elevation myocardial infarction; STEMI, ST‐segment elevation myocardial infarction.

### 3.2. MCG Parameter Selection

**Figure 2:**
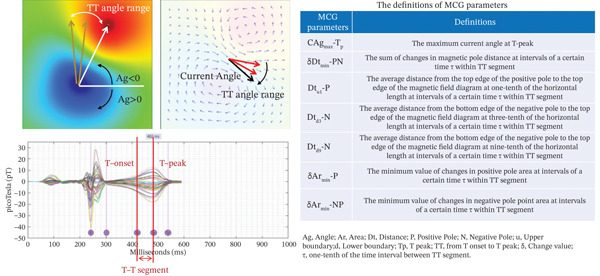
Schematic definition of selected MCG parameters. The selected MCG parameters were derived from magnetic field maps and pseudocurrent density maps during repolarization‐related phases. Angle‐, distance‐, and area‐related indices were extracted from the spatial distribution and temporal changes of positive and negative magnetic poles. These parameters were used to construct an exploratory MCG composite variable.

Seven exploratory MCG parameters were selected to derive the MCG composite variable: CAg_max_‐Tp, *δ*Dt_min_‐PN, Dt_u1_‐P, Dt_d3_‐N, Dt_d9_‐N, *δ*Ar_min_‐P, and *δ*Ar_min_‐NP (Figure 2). These parameters reflect spatial and temporal characteristics of the cardiac magnetic field and pseudocurrent density distributions during repolarization‐related phases. The parameters were standardized and combined into a single MCG composite variable using Cox regression. The full coefficients and effect estimates of the selected MCG parameters are provided in Table S2. Among the individual parameters, CAg_max_‐Tp showed the strongest association with MACCE, whereas the remaining parameters contributed to the composite score with variable effect sizes. The exploratory change in AUC with the cumulative addition of selected MCG parameters is summarized in Table S7 and Figure S2.

### 3.3. Cox Regression Analysis for Predicting MACCE

Univariable and multivariable Cox regression analyses were performed to evaluate predictors of MACCE (Table 2). In univariable analysis, chronic kidney disease (HR, 4.95; 95% CI, 1.75–13.99; *p* = 0.003), SYNTAX score (HR, 1.08; 95% CI, 1.04–1.12; *p* < 0.001), and the MCG composite variable (HR, 2.72; 95% CI, 1.57–4.70; *p* < 0.001) were associated with MACCE. In Model 2, which included clinical variables and SYNTAX score, SYNTAX score remained associated with MACCE (HR, 1.08; 95% CI, 1.04–1.12; *p* < 0.001). In Model 3, which further included the MCG composite variable, both SYNTAX score (HR, 1.08; 95% CI, 1.04–1.12; *p* < 0.001) and the MCG composite variable (HR, 2.74; 95% CI, 1.57–4.79; *p* < 0.001) were associated with MACCE. The full coefficients of the final Cox model are provided in Table S3. The global proportional hazard assumption was not violated for Model 3 (Table S4).

**Table 2:** Univariable and multivariable Cox regression analyses for MACCE.

Variable	Univariable		Model 1		Model 2		Model 3	
	HR (95% CI)	*p* value	HR (95% CI)	*p* value	HR (95% CI)	*p* value	HR (95% CI)	*p* value
Age	1.01 (0.98–1.05)	0.42	1.01 (0.97–1.05)	0.60	1.01 (0.97–1.04)	0.75	1.01 (0.97–1.05)	0.58
Male sex	0.83 (0.41–1.68)	0.61	0.99 (0.48–2.07)	0.98	0.92 (0.44–1.91)	0.81	1.10 (0.51–2.37)	0.80
BMI	0.98 (0.89–1.08)	0.67	0.99 (0.89–1.09)	0.78	0.99 (0.89–1.10)	0.84	0.97 (0.88–1.07)	0.51
Hypertension	1.51 (0.73–3.12)	0.27	1.44 (0.69–3.01)	0.33	1.56 (0.75–3.25)	0.24	1.46 (0.69–3.07)	0.32
Diabetes mellitus	1.59 (0.83–3.03)	0.16	1.53 (0.80–2.93)	0.20	1.41 (0.74–2.72)	0.30	1.40 (0.74–2.68)	0.30
Smoking history	0.68 (0.28–1.64)	0.39	0.73 (0.30–1.77)	0.49	0.68 (0.28–1.66)	0.39	0.62 (0.25–1.53)	0.30
Chronic kidney disease	4.95 (1.75–13.99)	0.003	—	—	—	—	—	—
SYNTAX score	1.08 (1.04–1.12)	< 0.001	—	—	1.08 (1.04–1.12)	< 0.001	1.08 (1.04–1.12)	< 0.001
MCG composite variable	2.72 (1.57–4.70)	< 0.001	—	—	—	—	2.74 (1.57–4.79)	< 0.001

*Note:* Model 1 included age, male sex, BMI, hypertension, diabetes mellitus, and smoking history. Model 2 included Model 1 plus the SYNTAX score. Model 3 included Model 2 plus the MCG composite variable. CKD was evaluated in a univariable analysis and additionally tested in a sensitivity model because of the low number of CKD cases.

Abbreviations: CI, confidence interval; HR, hazard ratio.

### 3.4. Model Performance and Internal Validation

**Figure 3:**
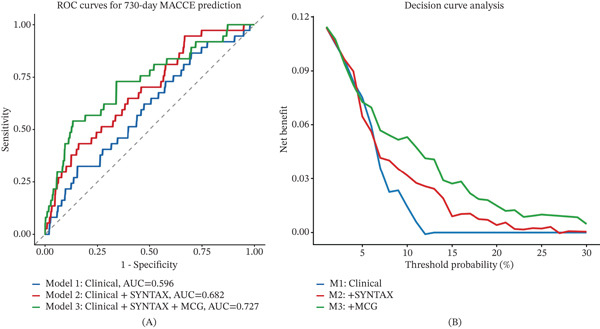
Model discrimination and decision curve analysis. (A) ROC curves for Model 1, Model 2, and Model 3. Model 1 included clinical variables only; Model 2 included clinical variables and SYNTAX score; and Model 3 included clinical variables, SYNTAX score, and the MCG composite variable. Model 3 showed numerically higher discrimination than Model 2, but the difference between Model 2 and Model 3 was not statistically significant. (B) Decision curve analysis of the three models across clinically relevant threshold probabilities.

The apparent AUC was 0.596 for Model 1, 0.682 for Model 2, and 0.727 for Model 3 (Table 3 and Figure 3A). Compared with Model 1, Model 3 showed significantly higher discrimination (*p* = 0.02). However, the improvement from Model 2 to Model 3 did not reach statistical significance (*p* = 0.17), indicating that the addition of the MCG composite variable resulted in a numerical, but not statistically significant, improvement in discrimination beyond clinical variables and SYNTAX score.

**Table 3:** Discrimination and calibration of the nested prognostic models.

Model	Variables included	Apparent ROC AUC	730‐day time‐dependent AUC (95% CI)	730‐day Brier score (95% CI)	*p* vs. preceding model	*p* vs. Model 1
Model 1	Clinical variables only	0.596	0.613 (0.518–0.708)	0.063 (0.045–0.082)	—	—
Model 2	Clinical variables + SYNTAX score	0.682	0.685 (0.597–0.774)	0.062 (0.044–0.079)	0.08	0.08
Model 3	Clinical variables + SYNTAX score + MCG composite	0.727	0.743 (0.654–0.833)	0.059 (0.042–0.076)	0.17	0.02

*Note:* Apparent ROC AUCs correspond to the ROC curves. Pairwise *p* values were calculated by DeLong′s test for apparent ROC AUC comparison. The 730‐day time‐dependent AUC and Brier score were estimated using survival data. Model 3 showed numerically higher discrimination and lower prediction error than Model 2, but the apparent ROC AUC improvement from Model 2 to Model 3 did not reach statistical significance.

At 730 days, the time‐dependent AUC increased from 0.613 for Model 1 to 0.685 for Model 2 and 0.743 for Model 3. The Brier score decreased from 0.063 for Model 1 to 0.062 for Model 2 and 0.059 for Model 3. Bootstrap internal validation showed an apparent *C*‐index of 0.719 and an optimism‐corrected *C*‐index of 0.672. Calibration assessment suggested acceptable overall calibration, although calibration remained imperfect in higher predicted‐risk ranges (Table S8 and Figure S3). Decision curve analysis showed that Model 3 provided numerically higher net benefit across part of the clinically relevant threshold range (Figure 3B). An exploratory nomogram based on Model 3 is provided in Figure S4.

### 3.5. Subgroup and Survival Analysis

**Figure 4:**
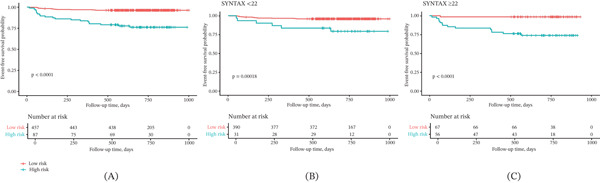
Kaplan–Meier curves according to the Youden‐derived Model 3 risk threshold. Patients were stratified into low‐ and high‐risk groups using the Youden‐derived cutoff of 10.5% predicted 730‐day MACCE risk from Model 3. (A) Overall cohort. (B) SYNTAX < 22 subgroup. (C) SYNTAX ≥ 22 subgroup. Subgroup analyses were exploratory.

The Youden‐derived cutoff for Model 3 predicted 730‐day MACCE risk was 10.5%. Using this cutoff, 457 patients were classified as low risk and 87 as high risk. MACCE occurred in 17 of 457 patients in the low‐risk group (3.72%) and in 20 of 87 patients in the high‐risk group (22.99%). The Kaplan–Meier analysis showed significantly lower event‐free survival in the high‐risk group than in the low‐risk group (log‐rank *p* < 0.001; Figure 4A).

Exploratory subgroup analyses were further performed according to SYNTAX score strata. In both the SYNTAX < 22 and SYNTAX ≥ 22 subgroups, patients classified as high risk by the Model 3‐derived cutoff had lower event‐free survival than those classified as low risk (Figure 4B,C). Given the limited number of events within subgroups, these analyses should be interpreted as exploratory.

### 3.6. Sensitivity Analyses

Because chronic kidney disease differed between patients with and without MACCE, an additional CKD‐adjusted sensitivity model was performed. In this model, CKD was associated with MACCE (HR, 4.95; 95% CI, 1.66–14.74; *p* = 0.004). SYNTAX score (HR, 1.08; 95% CI, 1.04–1.13; *p* < 0.001) and the MCG composite variable (HR, 2.68; 95% CI, 1.52–4.73; *p* < 0.001) remained associated with MACCE after further adjustment for CKD (Table S5). Penalized Cox regression retained SYNTAX score, chronic kidney disease, and selected MCG parameters at lambda.min, supporting the presence of an MCG‐related prognostic signal, although the more parsimonious lambda.1se model did not retain additional predictors (Table S6 and Figure S1).

## 4. Discussion

### 4.1. Main Findings

In this single‐center prospective cohort study of patients with 3V‐CAD undergoing revascularization, we evaluated whether MCG‐derived parameters could provide prognostic information for long‐term MACCE. The main findings were as follows. First, 37 of 544 patients experienced MACCE during a median follow‐up of 725 days. Second, SYNTAX score and the MCG composite variable were associated with MACCE after adjustment for clinical variables. Third, adding the MCG composite variable to the clinical + SYNTAX model numerically improved discrimination and reduced prediction error, although the improvement in AUC over the clinical + SYNTAX model did not reach statistical significance. Fourth, a Youden‐derived Model 3 risk threshold stratified MACCE risk in the overall cohort and in exploratory SYNTAX subgroups. Taken together, these findings suggest that MCG may provide complementary electrophysiological information for exploratory risk stratification in patients with 3V‐CAD. However, the results should be considered hypothesis‐generating because of the limited number of events, single‐center design, and lack of external validation.

### 4.2. Traditional Clinical Risk Factors for 3V‐CAD Patients

The condition of 3V‐CAD patients is relatively complex. Even after timely revascularization, choosing the appropriate treatment approach is crucial in the management of 3V‐CAD. Studies have found that age, the severity of chronic kidney disease, and triple‐vessel disease are independent predictors of 12‐month mortality after PCI. In patients with eGFR < 75, the use of DES can reduce the risk of MACCE, with an HR of 1.8 and *p* = 0.0044 [9]. Additionally, long‐term follow‐up of patients with triple‐vessel disease has revealed that stent implantation, hypertension, and eGFR or uric acid are independent predictors of MACCE in these patients, indicating that these factors need to be given special attention in drug treatment management [10]. Therefore, conducting prognostic risk stratification for patients with triple‐vessel disease can better manage their follow‐up treatment after surgery.

### 4.3. Risk Stratification by SYNTAX Score

Regarding the risk assessment of the prognosis for 3V‐CAD patients, the SYNTAX score can be used to predict the MACCE after PCI. In the substudy of FAME3 [11], it was found that among the patients randomly assigned to receive PCI, those with a lower SYNTAX score value (≤ 22) had a similar MACCE rate at 1 year compared to those who received CABG treatment. After adding the functional FFR parameters, the functional‐based SYNTAX score was an independent predictor of major adverse coronary events in patients randomly assigned to receive PCI [11]. In the study by Kobayashi et al., the improved scoring based on SYNTAX, such as the residual SYNTAX score (RSS) and SYNTAX revascularization index (SRI), demonstrated reliable efficacy in quantitatively assessing the revascularization integrity as shown by angiography in patients with multivessel CAD [12, 13]. Similarly, adding the SYNTAX score improved model discrimination, and further addition of the MCG composite variable resulted in numerical improvement in discrimination and prediction error. Our previous work was conducted using the same OPM‐MCG platform and should therefore be interpreted as platform‐specific preliminary evidence rather than independent external validation. The present study extends this previous work by exploring whether MCG‐derived electrophysiological information is associated with long‐term outcomes in patients with 3V‐CAD.

### 4.4. Risk Stratification by MCG

MCG is a noninvasive, noncontact, and radiation‐free technique for recording the electromagnetic activity of the heart. MCG is particularly sensitive to the tangential currents and circular eddies generated during myocardial ischemia and can accurately capture the subtle electrophysiological changes of the heart [6, 14]. Previous studies have confirmed the clinical value of MCG in detecting myocardial ischemia. The earliest research conducted by Park et al. showed that the sensitivity and specificity of MCG in diagnosing coronary heart disease were 95.1% and 92.8%, respectively [15]. Our team′s previous research also confirmed that the machine learning model based on MCG parameters can predict myocardial perfusion injury, and this conclusion has been verified by single‐photon emission computed tomography (SPECT), indicating that MCG has potential in detecting myocardial perfusion injury [16]. In addition, the team previously developed a myocardial ischemia prediction model for patients with severe CAD based on MCG parameters, with an AUC value of 0.864, indicating that this model can assess the myocardial ischemia status of patients before invasive coronary angiography, thereby reducing unnecessary angiography examinations [17]. All these findings suggest that MCG has the potential to become a tool for risk stratification of prognosis in patients with 3V‐CAD.

In the present study, adding the MCG composite variable to clinical variables and SYNTAX score resulted in numerical improvement in model discrimination and prediction error. However, the improvement over the clinical + SYNTAX model did not reach statistical significance, and the findings should therefore be interpreted as exploratory. From a mechanistic perspective, MCG may capture electrophysiological heterogeneity related to myocardial ischemia or repolarization abnormalities that is not directly reflected by anatomical lesion complexity. Thus, MCG may serve as a complementary tool rather than a replacement for established anatomical or functional assessments. Further multicenter studies with external validation are required to determine whether MCG provides clinically meaningful complementary prognostic value in patients with 3V‐CAD.

### 4.5. Positioning of MCG Relative to Established Noninvasive Ischemia Tests

The potential role of MCG should be interpreted in the context of established noninvasive strategies for ischemia detection and risk stratification. Conventional functional tests, including exercise stress testing, stress echocardiography, myocardial perfusion imaging, and stress cardiac magnetic resonance, evaluate inducible ischemia through different physiological or imaging targets. A recent systematic review and meta‐analysis comparing these modalities suggested that stress CMR had the highest overall diagnostic accuracy, whereas stress echocardiography provided a balanced option considering diagnostic performance, availability, cost‐effectiveness, and absence of ionizing radiation [18]. In clinical practice, stress echocardiography may be limited by operator dependence and image quality [19], SPECT provides established perfusion‐based diagnostic and prognostic information but involves radiation exposure and potential attenuation artifacts [20], and stress CMR offers a comprehensive assessment without radiation but may be restricted by availability, cost, acquisition time, and contraindications [21]. Coronary CT angiography remains an important first‐line anatomical test in patients with low‐to‐moderate pretest likelihood, whereas functional imaging is often preferred when ischemia or viability assessment is required [22].

Unlike these anatomy‐ or perfusion‐centered modalities, MCG captures cardiac magnetic field abnormalities related to myocardial electrophysiological heterogeneity. Therefore, MCG should not be considered a replacement for established stress imaging or CT‐based strategies but rather a potential complementary tool that may add electrophysiological information to anatomical risk assessment in selected high‐risk patients such as those with 3V‐CAD. In the present study, the addition of the MCG composite variable numerically improved model discrimination and reduced prediction error, but its incremental value beyond clinical variables and the SYNTAX score requires further confirmation in larger multicenter cohorts with external validation.

### 4.6. Limitation

Several limitations should be acknowledged. First, this was a single‐center prospective observational cohort study conducted at a tertiary cardiovascular center, and selection bias cannot be excluded. The cohort was restricted to revascularization‐naïve patients with 3V‐CAD who underwent PCI or CABG; therefore, the findings may not be generalizable to patients with prior revascularization, medically managed CAD, lower risk CAD populations, or broader real‐world settings. Second, the number of observed MACCE events was limited. Although bootstrap internal validation, calibration assessment, Brier score analysis, and penalized Cox sensitivity analysis were performed, the model remains vulnerable to overfitting and should be considered exploratory until externally validated. Third, the selected MCG parameters are not yet standardized clinical biomarkers, and the MCG composite variable was derived using a single OPM‐MCG platform. Therefore, the generalizability of the selected parameters and risk model to other MCG systems remains uncertain. Fourth, MACCE included unplanned revascularization, which may be influenced by local clinical practice patterns. Because the number of component events was limited, especially for cardiac death and other hard endpoints, robust competing‐risk modeling could not be performed. Finally, subgroup analyses according to SYNTAX strata were exploratory and underpowered. Larger multicenter studies with external validation, longer follow‐up, standardized MCG acquisition protocols, and detailed component endpoint adjudication are needed to confirm the prognostic utility and clinical applicability of MCG.

## 5. Conclusion

In this single‐center prospective cohort study of patients with 3V‐CAD undergoing revascularization, the MCG composite variable was associated with long‐term MACCE after adjustment for clinical variables and SYNTAX score. Adding MCG numerically improved model discrimination and reduced prediction error, although the improvement over the clinical + SYNTAX model was not statistically significant. These findings support the potential role of MCG as an exploratory complementary risk stratification tool. Larger multicenter studies with external validation are required to confirm its complementary prognostic value and clinical applicability.

## Author Contributions

Hongjia Zhang, Xiantao Song, and Chenchen Tu contributed to the conception of the topic. Ziyu An and Shipan Wang wrote the manuscript and conducted the statistical analysis. Shuwen Yang and Lanxin Feng performed the data collection and collation. Min Zhang and Mingduo Zhang helped to finish the statistics. Qi Zhou and Haiyang Hu helped to structure the text. Hongjia Zhang and Chenchen Tu helped to revise the manuscript. Ziyu An and Shipan Wang contributed equally to this work and share the first authorship

## Funding

This study was funded by the Capital′s Funds for Health Improvement and Research (2024‐2‐2066), the Coordinated Innovation of Scientific and Technological in the Beijing‐Tianjin‐Hebei Region (Z231100003923008), the Beijing Nova Program (10.13039/501100005090) (20220484222), the Project of the Beijing Lab for Cardiovascular Precision Medicine (PXM2018_014226_000013), the High‐Level Public Health Technical Talent Construction Project of the Beijing Municipal Health Commission (Leading Talent‐02‐01), and the Beijing Hospitals Authority′s Ascent Plan (DFL20220603).

## Disclosure

All authors have read and approved the final version of the manuscript. The authors had full access to all of the data in this study and take complete responsibility for the integrity of the data and the accuracy of the data analysis. Chenchen Tu affirms that this manuscript is an honest, accurate, and transparent account of the study being reported; that no important aspects of the study have been omitted; and that any discrepancies from the study as planned have been explained. This manuscript uses an overlapping source cohort from a previously published study [23]. The previous publication addressed the clinician‐selected revascularization category, whereas the present study focuses on long‐term MACCE risk stratification after revascularization.

## Ethics Statement

This study is a prospective, single‐center, observational cohort study. The study protocol has been reviewed by the Ethics Committee of Beijing Anzhen Hospital, Capital Medical University. All participants signed the informed consent form.

## Conflicts of Interest

The authors declare no conflicts of interest.

## General Statement

This manuscript was prepared in accordance with the STROBE recommendations for observational cohort studies and incorporated relevant TRIPOD reporting elements for prognostic model development and evaluation.

## Supporting information


**Supporting Information** Additional supporting information can be found online in the Supporting Information section. Table S1 summarizes missing data for variables included in the primary analyses. Table S2 provides definitions and effect estimates of the selected MCG parameters. Table S3 lists the full coefficients of the final Cox model. Table S4 reports proportional hazard assumption testing for the final Cox model. Table S5 presents the CKD‐adjusted sensitivity Cox model. Table S6 summarizes the LASSO‐penalized Cox sensitivity analysis. Table S7 describes the exploratory AUC change with cumulative MCG parameters. Table S8 presents the calibration of Model 3 by predicted‐risk groups. Figure S1 shows the cross‐validation curve for LASSO‐penalized Cox regression. Figure S2 shows the exploratory AUC change with cumulative MCG parameters. Figure S3 shows the calibration plot for Model 3. Figure S4 presents the exploratory nomogram based on Model 3.

## Data Availability

The data that support the findings of this study are available from the corresponding authors upon reasonable request.
