# Supplementary material for: Magnetocardiography for Exploratory Risk Stratification in Patients With Three‐Vessel Coronary Artery Disease: A Single‐Center Prospective Cohort Study
Source: Cardiovasc Ther. 2026 Jul 16;2026:5552371. doi: 10.1155/cdr/5552371 (PMC13376331; doi:10.1155/cdr/5552371)
Supplement: Supplementary file 1 — Supporting Information Additional supporting information can be found online in the Supporting Information section. Table S1 summarizes missing data for variables included in the primary analyses. Table S2 provides definitions and effect estimates of the selected MCG parameters. Table S3 lists the full coefficients of the final Cox model. Table S4 reports proportional hazard assumption testing for the final Cox model. Table S5 presents the CKD‐adjusted sensitivity Cox model. Table S6 summarizes the LASSO‐penalized Cox sensitivity analysis. Table S7 describes the exploratory AUC change with cumulative MCG parameters. Table S8 presents the calibration of Model 3 by predicted‐risk groups. Figure S1 shows the cross‐validation curve for LASSO‐penalized Cox regression. Figure S2 shows the exploratory AUC change with cumulative MCG parameters. Figure S3 shows the calibration plot for Model 3. Figure S4 presents the exploratory nomogram based on Model 3. [file CDR-2026-5552371-s001.docx]

**Supplementary Tables**

**Supplementary Table S1. Missing-data summary for variables included in the primary analyses.**

| **Variable** | **Missing, n** | **Total, n** | **Missing, %** |
| --- | --- | --- | --- |
| MACCE | 0 | 544 | 0 |
| Follow-up time, days | 0 | 544 | 0 |
| Age | 0 | 544 | 0 |
| Sex | 0 | 544 | 0 |
| BMI | 0 | 544 | 0 |
| Hypertension | 0 | 544 | 0 |
| Diabetes mellitus | 0 | 544 | 0 |
| Smoking history | 0 | 544 | 0 |
| Chronic kidney disease | 0 | 544 | 0 |
| LVEF | 0 | 544 | 0 |
| STEMI | 0 | 544 | 0 |
| NSTEMI | 0 | 544 | 0 |
| Unstable angina | 0 | 544 | 0 |
| Stable angina | 0 | 544 | 0 |
| Peripheral arterial disease | 0 | 544 | 0 |
| Hyperlipidaemia | 0 | 544 | 0 |
| SYNTAX score | 0 | 544 | 0 |
| CAgmax-Tp | 0 | 544 | 0 |
| δDtmin-PN | 0 | 544 | 0 |
| Dtu1-P | 0 | 544 | 0 |
| Dtd3-N | 0 | 544 | 0 |
| Dtd9-N | 0 | 544 | 0 |
| δArmin-P | 0 | 544 | 0 |
| δArmin-NP | 0 | 544 | 0 |

Note: Missingness was assessed for variables included in the primary analyses. No imputation was performed because no missing values were present for these variables.

**Supplementary Table S2. Definitions and effect estimates of the selected MCG parameters.**

| **Parameter** | **Source map** | **Parameter domain** | **Definition** | **β coefficient** | **SE** | **HR (95% CI)** | **P value** |
| --- | --- | --- | --- | --- | --- | --- | --- |
| CAgmax-Tp | Pseudo-current density map | Current angle | Current-angle parameter derived from the maximum positive pseudo-current distribution during the T-wave peak. | -0.4163 | 0.1631 | 0.66 (0.48–0.91) | .01 |
| δDtmin-PN | Magnetic field / pole-distance map | Distance change | Change in the minimum distance-related parameter between positive and negative magnetic poles. | 0.1453 | 0.2236 | 1.16 (0.75–1.79) | .52 |
| Dtu1-P | Magnetic field / pole-distance map | Distance | Distance-related parameter derived from the positive magnetic pole distribution during the T-wave phase. | -1.1866 | 1.3073 | 0.31 (0.02–3.96) | .36 |
| Dtd3-N | Magnetic field / pole-distance map | Distance | Distance-related parameter derived from the negative magnetic pole distribution during the T-wave phase. | -0.2885 | 0.1990 | 0.75 (0.51–1.11) | .15 |
| Dtd9-N | Magnetic field / pole-distance map | Distance | Distance-related parameter derived from the negative magnetic pole distribution during the T-wave phase. | -0.3448 | 0.2524 | 0.71 (0.43–1.16) | .17 |
| δArmin-P | Magnetic field / area map | Area change | Change in the minimum area-related parameter of the positive magnetic pole. | 0.1350 | 0.2050 | 1.14 (0.77–1.71) | .51 |
| δArmin-NP | Magnetic field / area map | Area change | Change in the minimum area-related parameter between negative and positive magnetic poles. | -0.0931 | 0.1474 | 0.91 (0.68–1.22) | .53 |

Note: The selected parameters were standardized before Cox modeling. Definitions refer to device-derived exploratory MCG indices and should be interpreted as components of the MCG composite variable rather than standalone clinical biomarkers. CI, confidence interval; MCG, magnetocardiography; SE, standard error.

**Supplementary Table S3. Full coefficients of the final Cox model.**

| **Predictor** | **β coefficient** | **SE** | **HR (95% CI)** | **P value** |
| --- | --- | --- | --- | --- |
| Age | 0.0102 | 0.0184 | 1.01 (0.97–1.05) | .58 |
| Male sex | 0.0998 | 0.3900 | 1.10 (0.51–2.37) | .80 |
| BMI | -0.0333 | 0.0508 | 0.97 (0.88–1.07) | .51 |
| Hypertension | 0.3782 | 0.3798 | 1.46 (0.69–3.07) | .32 |
| Diabetes mellitus | 0.3396 | 0.3298 | 1.40 (0.74–2.68) | .30 |
| Smoking history | -0.4730 | 0.4584 | 0.62 (0.25–1.53) | .30 |
| SYNTAX score | 0.0752 | 0.0198 | 1.08 (1.04–1.12) | <.001 |
| MCG composite variable | 1.0071 | 0.2851 | 2.74 (1.57–4.79) | <.001 |

Note: The final model included clinical variables, SYNTAX score, and the MCG composite variable. CI, confidence interval; MCG, magnetocardiography; SE, standard error.

**Supplementary Table S4. Proportional hazards assumption testing for the final Cox model.**

| **Variable** | **χ²** | **df** | **P value** |
| --- | --- | --- | --- |
| Age | 0.087 | 1 | .77 |
| Male sex | 1.192 | 1 | .27 |
| BMI | 0.182 | 1 | .67 |
| Hypertension | 4.380 | 1 | .04 |
| Diabetes mellitus | 0.288 | 1 | .59 |
| Smoking history | 2.889 | 1 | .09 |
| SYNTAX score | 0.390 | 1 | .53 |
| MCG composite variable | 0.008 | 1 | .93 |
| Global test | 9.349 | 8 | .31 |

Note: Proportional hazards assumptions were assessed using Schoenfeld residuals. The global test was not statistically significant.

**Supplementary Table S5. CKD-adjusted sensitivity Cox model.**

| **Predictor** | **β coefficient** | **SE** | **HR (95% CI)** | **P value** |
| --- | --- | --- | --- | --- |
| Age | 0.0158 | 0.0181 | 1.02 (0.98–1.05) | .38 |
| Male sex | 0.0354 | 0.3916 | 1.04 (0.48–2.23) | .93 |
| BMI | -0.0264 | 0.0502 | 0.97 (0.88–1.07) | .60 |
| Hypertension | 0.3087 | 0.3795 | 1.36 (0.65–2.87) | .42 |
| Diabetes mellitus | 0.2664 | 0.3336 | 1.31 (0.68–2.51) | .42 |
| Smoking history | -0.5396 | 0.4634 | 0.58 (0.24–1.45) | .24 |
| Chronic kidney disease | 1.5985 | 0.5570 | 4.95 (1.66–14.74) | .004 |
| SYNTAX score | 0.0786 | 0.0203 | 1.08 (1.04–1.13) | <.001 |
| MCG composite variable | 0.9874 | 0.2885 | 2.68 (1.52–4.73) | <.001 |

Note: Chronic kidney disease was added to the final model as a sensitivity analysis because it differed between patients with and without MACCE at baseline. CI, confidence interval; CKD, chronic kidney disease; MACCE, major adverse cardiovascular and cerebrovascular events; MCG, magnetocardiography; SE, standard error.

**Supplementary Table S6. LASSO penalized Cox sensitivity analysis.**

| **Lambda** | **Lambda value** | **Selected variable** | **Coefficient** |
| --- | --- | --- | --- |
| lambda.min | 0.01228 | Hypertension | 0.0017 |
| lambda.min | 0.01228 | Diabetes mellitus | 0.0383 |
| lambda.min | 0.01228 | Smoking history | -0.0045 |
| lambda.min | 0.01228 | Chronic kidney disease | 1.2982 |
| lambda.min | 0.01228 | SYNTAX score | 0.0564 |
| lambda.min | 0.01228 | CAgmax-Tp | -0.1604 |
| lambda.min | 0.01228 | Dtu1-P | -0.0306 |
| lambda.1se | 0.04117 | No variable retained |  |

Note: Penalized Cox regression was performed using LASSO regularization. The lambda.min model retained selected predictors, whereas the more parsimonious lambda.1se model retained no additional predictors.

**Supplementary Table S7. Exploratory AUC change with cumulative MCG parameters.**

| **Number of cumulative MCG parameters** | **Added parameter** | **AUC** |
| --- | --- | --- |
| 1 | CAgmax-Tp | 0.565 |
| 2 | δDtmin-PN | 0.594 |
| 3 | Dtu1-P | 0.610 |
| 4 | Dtd3-N | 0.636 |
| 5 | Dtd9-N | 0.658 |
| 6 | δArmin-P | 0.650 |
| 7 | δArmin-NP | 0.669 |

Note: This exploratory analysis describes the change in AUC when selected MCG parameters were cumulatively added. It should not be interpreted as external validation. AUC, area under the receiver operating characteristic curve; MCG, magnetocardiography.

**Supplementary Table S8. Calibration of Model 3 by predicted-risk groups.**

| **Calibration group** | **n** | **Events** | **Mean predicted risk, %** | **Observed risk, %** |
| --- | --- | --- | --- | --- |
| 1 | 109 | 3 | 1.66 | 2.75 |
| 2 | 109 | 3 | 3.35 | 2.75 |
| 3 | 109 | 4 | 4.96 | 3.70 |
| 4 | 109 | 7 | 7.27 | 6.42 |
| 5 | 108 | 20 | 17.58 | 19.05 |

Note: Patients were grouped into five strata according to predicted 730-day MACCE risk from Model 3. Observed risk was estimated using the Kaplan-Meier method. MACCE, major adverse cardiovascular and cerebrovascular events.

**Supplementary Figure**

**Supplementary Figure S1. Cross-validation curve for LASSO penalized Cox regression.**


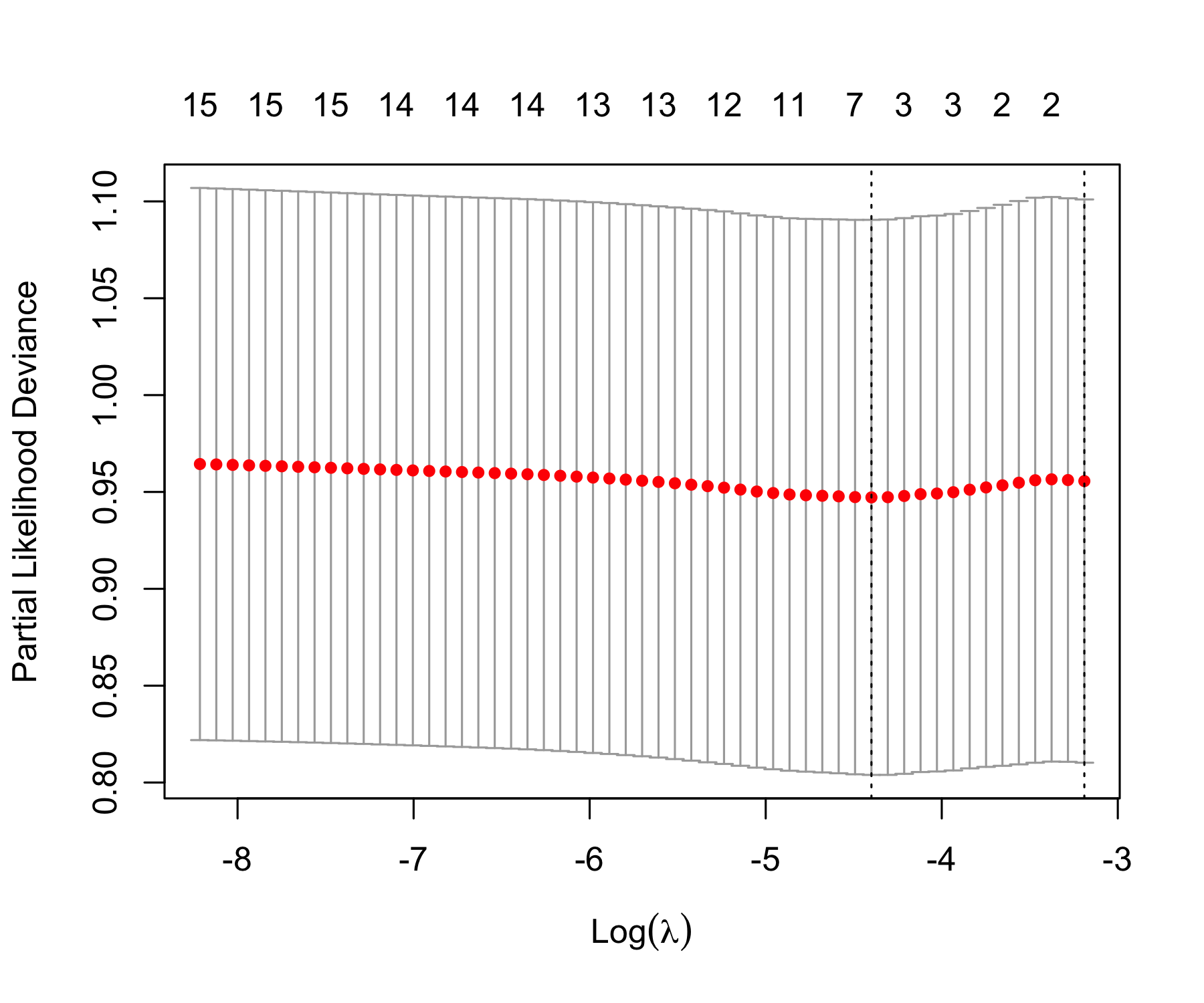


Cross-validation curve for the LASSO penalized Cox regression sensitivity analysis. The x-axis represents log-transformed lambda values, and the y-axis represents the partial likelihood deviance. Red points indicate the mean cross-validated partial likelihood deviance, and grey vertical bars indicate the corresponding standard errors. The vertical dashed lines indicate the lambda.min and lambda.1se values. Numbers along the upper axis indicate the number of non-zero coefficients retained at each lambda value. This analysis was performed to assess potential overfitting and variable-selection instability in the exploratory prognostic model.

**Supplementary Figure S2. Exploratory AUC change with cumulative MCG parameters.**

**
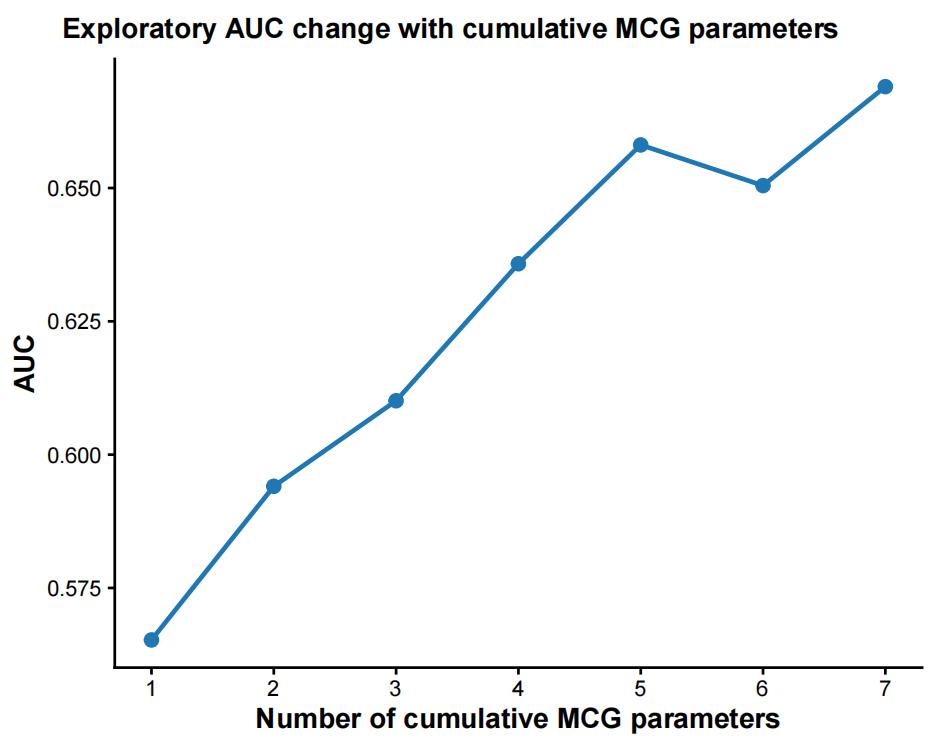
**

Exploratory change in the area under the receiver operating characteristic curve with cumulative addition of selected MCG parameters. The selected MCG parameters were added sequentially in the following order: CAgmax-Tp, δDtmin-PN, Dtu1-P, Dtd3-N, Dtd9-N, δArmin-P, and δArmin-NP. The AUC increased from 0.565 with the first parameter to 0.669 after inclusion of all seven selected MCG parameters. This analysis was exploratory and should not be interpreted as external validation of the MCG composite variable.

**Supplementary Figure S3. Calibration plot for Model 3.**

**
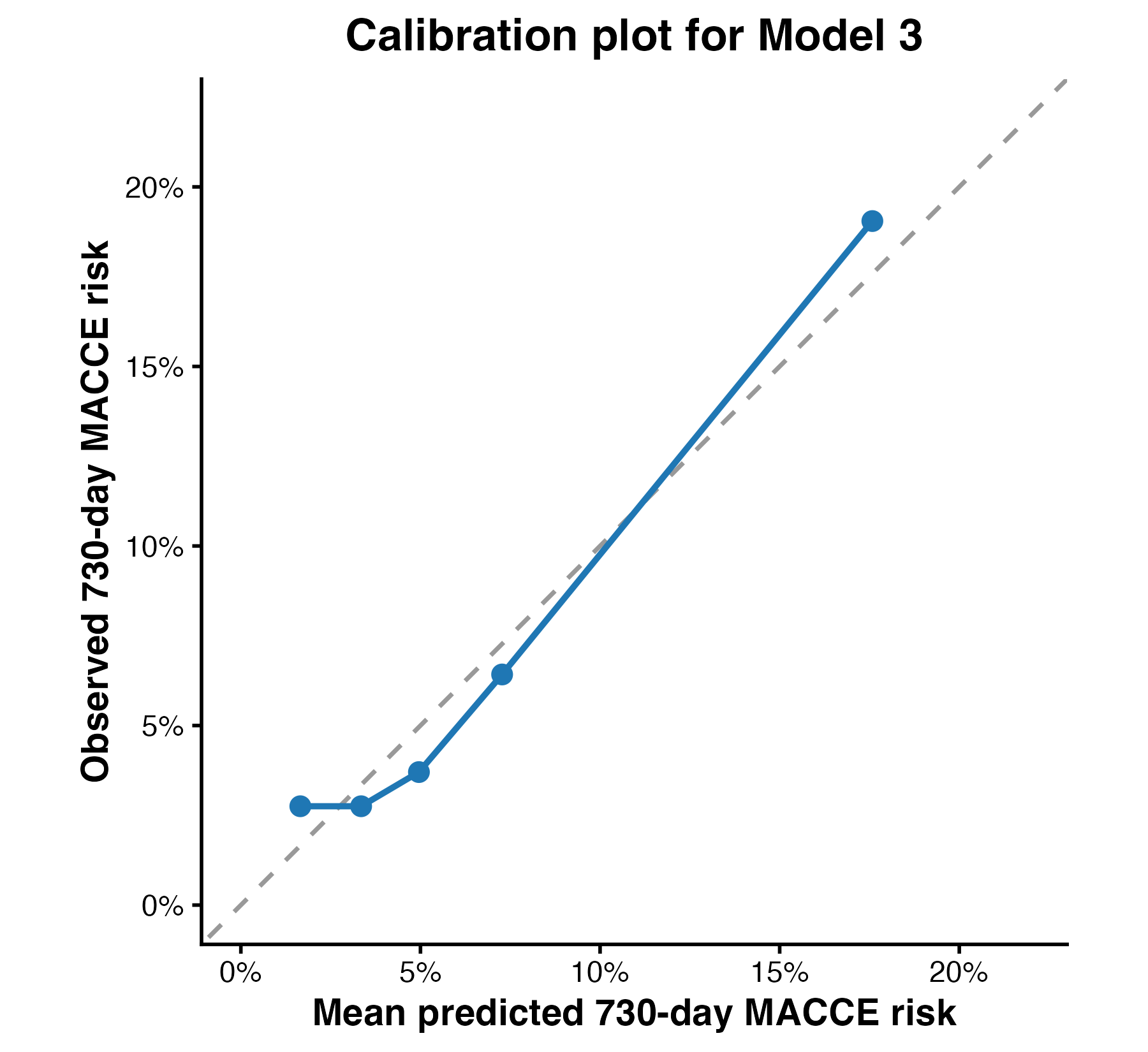
**

Calibration plot for Model 3 predicting 730-day MACCE risk. The x-axis represents the mean predicted 730-day MACCE risk, and the y-axis represents the observed 730-day MACCE risk estimated across predicted-risk groups. The dashed diagonal line represents perfect calibration. Model 3 showed acceptable overall agreement between predicted and observed risk, although calibration remained imperfect in higher predicted-risk ranges. MACCE, major adverse cardiovascular and cerebrovascular events.

**Supplementary Figure S4. Exploratory nomogram based on Model 3.**

**
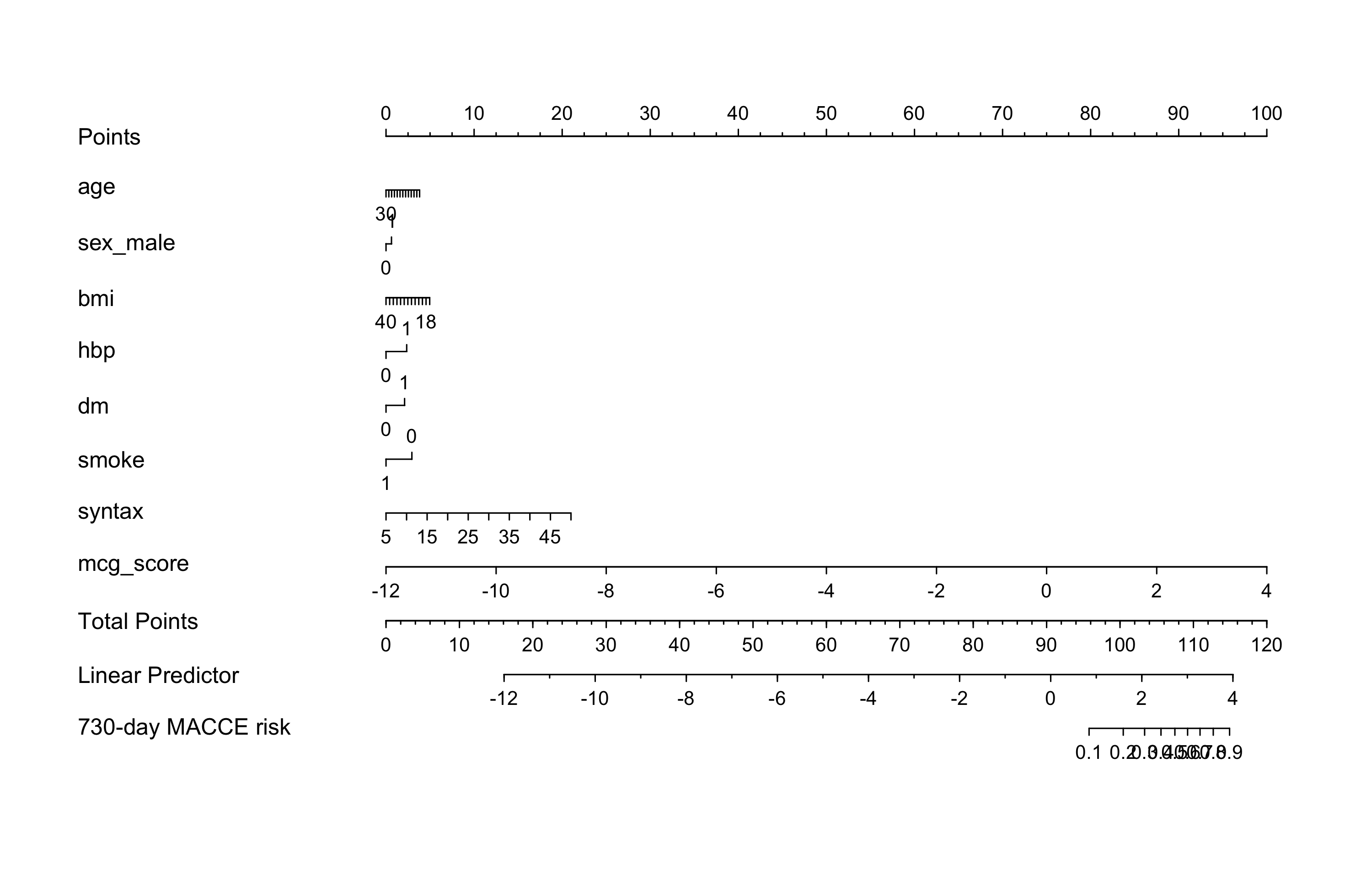
**

Exploratory nomogram based on Model 3 for estimating 730-day MACCE risk in patients with three-vessel coronary artery disease after revascularization. Model 3 included clinical variables, SYNTAX score, and the MCG composite variable. Points assigned to each predictor are summed to obtain a total point score, which corresponds to the estimated 730-day MACCE risk. This nomogram was developed for exploratory visualization of the prognostic model and requires external validation before clinical application. MACCE, major adverse cardiovascular and cerebrovascular events; MCG, magnetocardiography.
